# Supplementary material for: WisecondorFF: Improved Fetal Aneuploidy Detection from Shallow WGS through Fragment Length Analysis
Source: Diagnostics (Basel). 2021 Dec 28;12(1):59. doi: 10.3390/diagnostics12010059 (PMC8774687; doi:10.3390/diagnostics12010059)
Supplement: Supplementary file 1 [file diagnostics-12-00059-s001.zip › diagnostics-1509027-supplementary.pdf]

Supplemental Materials

1. Processing and filtering paired-end reads

When pre-processing, WisecondorFF discretizes the genome and accumulates the read coverage together with the fragment size statistics of each sample. During this accumulation process paired-end reads are subject to filtering based on the following criteria: (1) reads must be in the correct position/orientation for pairing; (2) only primary alignments are considered; (3) alignments must exceed a minimum mapping quality of at least 1; (4) every read should have a unique starting location. In (1) we consider that only proper pairing should be considered for further analysis, i.e. when pairing fails we exclude such reads. With (2) and (3) we account and filter for reads that map to repetitious regions of the genome, hence there being multiple valid mappings of a read, which are then given a mapping quality score of 0. Lastly with (4) we exclude any reads that were found to map to locations that were previously aligned to. Within the low yield WGS (~0.25x coverage) setting (expecting an approximate uniform genome mapping distribution), it is unexpected for many reads to map to the exact same location. In Figure S1a we show the coverage distribution of chromosome 1 within a single sample, in which (4) filtering was not performed. Right at the centromere boundary a large pileup is concentrated caused by (technical) duplication, which would adversely affect reference set construction. It is trivial to account for this duplication effect by using the (4) filtering, as can be observed in Figure S1b.

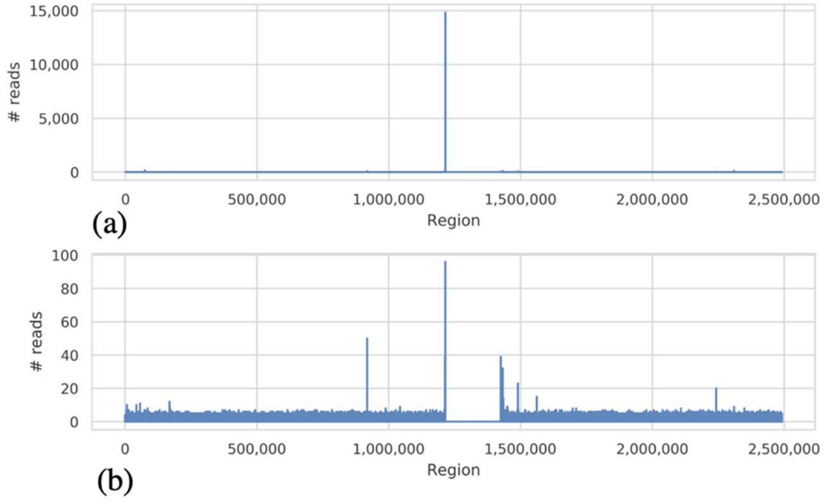

Figure S1. 100 bp region stratified read coverage (~0.25x) of chromosome 1 in a single sample. **(a)** Prior to filtering out reads beginning at the exact same genomic location. **(b)** Post-filtering, removes such duplicated reads.

2. Filtering regions by depth of coverage

Any sample processed by WisecondorFF is subject to initial filtering. Such filtering excludes regions (defined after discretization) that contain insufficient information, i.e. too few reads are aligned within these genomic regions. In such cases these regions cannot be confidently utilized as reference regions, meaning CNV calling is also not possible at these locations. Although the fragment size is utilized, we base region filtering on the read coverage (the fragment size is also derived from aligned reads). In our experiments we found that any region should contain at least 500 reads for it to be sufficiently informative. This cutoff hinges directly on the average depth of coverage and chosen region size, where a smaller chosen region size lowers the probability that sufficient reads are contained in any region. In Figure S2a and S2b we consider the genome-wide per-region coverage of two samples with different depths of coverage, with a different cutoff for each distribution. Note that the majority of regions contain sufficient read, but that there is a small proportion where (almost) no reads are aligned, which are the regions that should be filtered out. A constant size filtering is not adequate given that every sample has a different depth of coverage, Figure S2c. Meaning that a threshold may be overly aggressive in a particular sample, e.g. a cutoff of 1500 is proper for SAMPLE_2 but too aggressive for SAMPLE_1. To account for this we utilize a secondary filtering based on the normalized coverage, Figure S2d. Which allows for an uniform cutoff across all samples regardless of coverage.

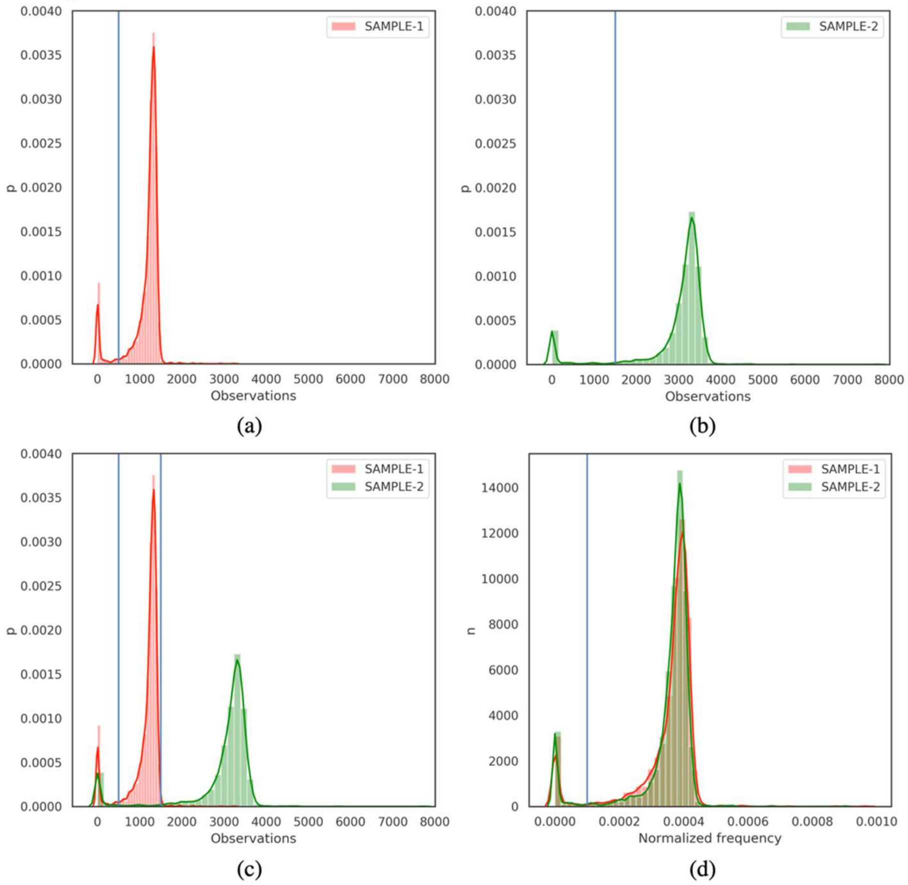

Figure S2. Per-region coverage distribution plots: **(a)** Sample with an optimal cutoff at 500 reads. **(b)** Sample with an optimal cutoff at 1500 reads. **(c)** Both samples overlaid showing both cutoffs. **(d)** Both samples are overlaid with normalized frequencies showing a cutoff of 0.0001.

3. Fragment size with respect to GC content and read count

The pre-processing of samples includes correction to account for bias caused by effects such as GC content. The effects on read count due to GC content are well known (Figure S3), and can be corrected for easily with LOWESS or PCA, where we used the latter. Although the fragment/insert size is independent from the GC content, we found that PCA correction still accounted for other types of bias within the fragment size data.

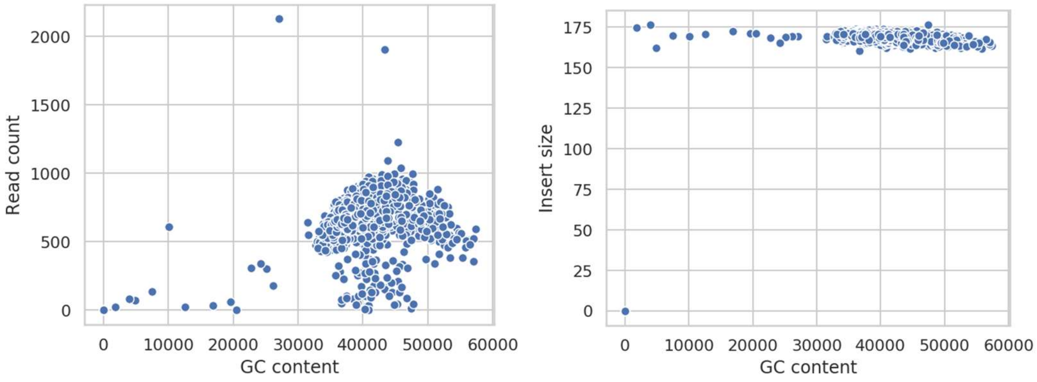

Figure S3. Genome-wide GC content with respect to the read count and the fragment/insert size respectively.

4. The fragment size median is less predictive than the mean

The per region fragment size statistics may be utilized in different ways, e.g. by directly comparing them by treating them as probability distributions as in Supplemental Section 7 or by further summarizing them using measures such as the mean or median. Practically we found that the per region fragment size mean was most predictive compared to other forms of summarization, with the median being the less effective secondary choice. Singular value metrics such as the mean or median can be used interchangeably, under the assumption that a distance metric may be calculated between any two values. Hence it was trivial to experiment with different summarization metrics and evaluate their performance. Figure S4 shows the average Z-scores across chromosome 21 for each of the 526 samples for regions of 1 Mb size comparing WcrFF^{RC}, the modus of WisecondorFF that only utilizes the read count, and WcrFF$^{RC\&FSmedian}$, which combines the read count and the fragment size median. Relative to the results we obtained, in Results 3.3 Figure 4, the fragment size median, while predictive, is not competitive with the mean.

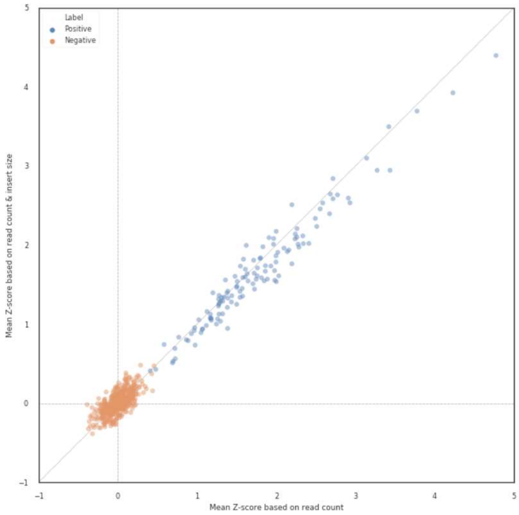

Figure S4. The average Z-score of all regions (1 Mb size) on chromosome 21 for all 526 (positive and negative) samples comparing WcrFF^{RC} and WcrFF$^{RC\&FSmedian}$.

5. Fetal fraction estimation with SeqFF

The fetal fraction plays an integral role within NIPT screening where if too little fetal DNA is present in the maternal serum, no reliable DNA-based testing is possible. In general the expectation is that test reliability increases as the fetal DNA concentration increases, since the fetal signal then becomes stronger. Relating the fetal fraction with method performance, e.g. based on sensitivity or robustness, is useful since it is important that any method does well at any given fetal fraction. Hence it is one approach of distinguishing and ranking method performance. To determine the fetal fraction we utilized SeqFF, a method that utilizes the aligned read counts in specific autosomal regions applying a weighing scheme derived from a pre-trained multivariate model. This model was trained on the per region stratified autosomal read counts from WGS paired-end sequencing of cfDNA in maternal plasma. In Figure S5 we summarize the stratified fetal fraction distributions of all 526 samples.

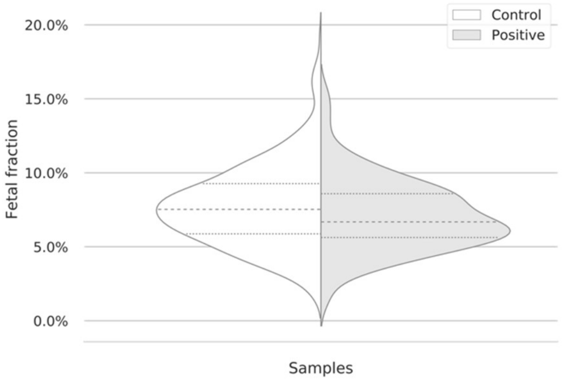

Figure S5. Fetal fraction values for all 526 samples as estimated by SeqFF. Samples are grouped accordingly to 401 healthy controls and 125 T21 positives.

6. Fragment size distribution estimation

The per sample fragment size statistics may be discretized and processed on different scales. The choice of scale is a direct trade-off in resolution and noise, dependent on the sample depth of coverage. With smaller region sizes there may be too much noise or too few reads available to accurately reconstruct the fragment size distribution. For the purpose of within-sample testing it is crucial that (nearly) all regions have sufficient measurements to be reliably used as reference regions. Hence it is important to determine the lower bound at which fragment size distribution estimation becomes robust, meaning there is no instability on any region across the genome within a sample. In Figure S6 we summarize the per region fragment size distribution of chromosome 1 in terms of the mean and standard deviation. Region sizes below 250 kb display increased variability or even missing values within the region fragment size distribution. Meaning a minimum region size of 250 kb is necessary to reliably estimate the fragment size distributions, which we further verified by testing distribution normality and distance from the expected mean and standard deviation of each region compared to a reference distribution and different samples.

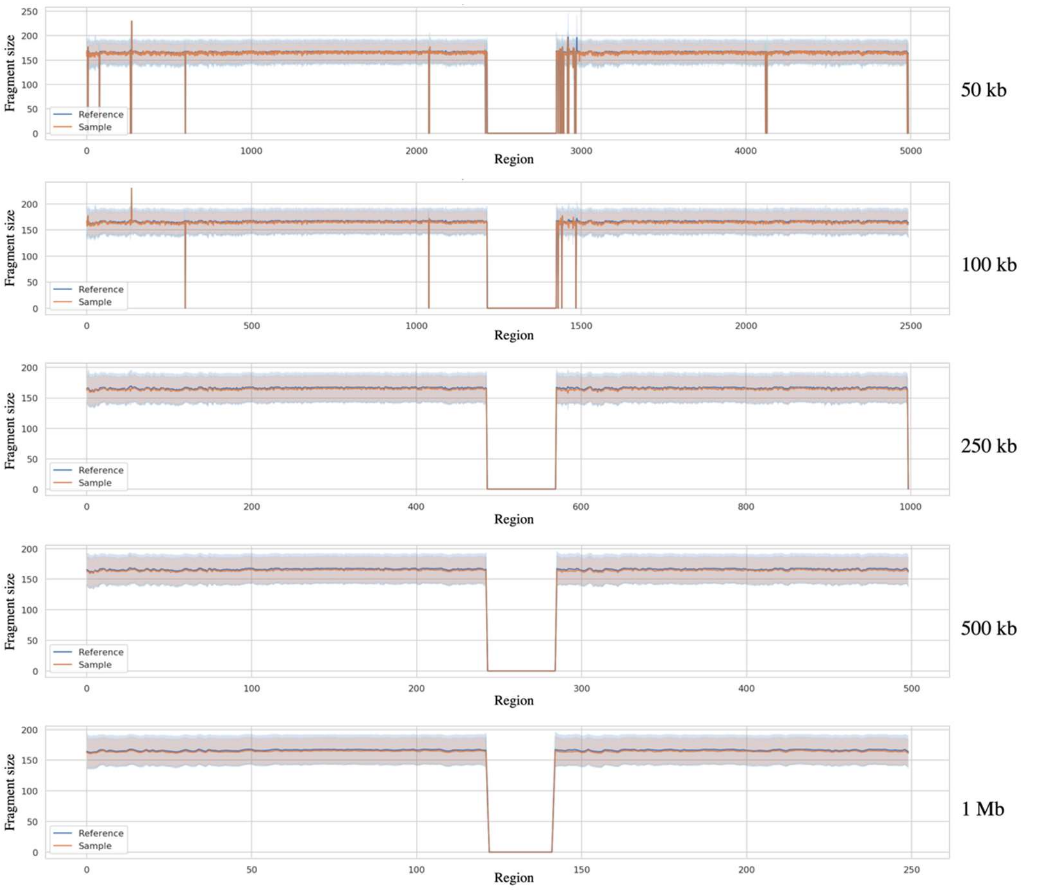

Figure S6. Per region fragment size distribution of chromosome 1 as summarized by the mean and standard deviation across different region scales. The orange colored line denotes a single sample, whereas the blue line is an aggregate of a collection of samples, used here to compare to as a baseline.

7. Reference set construction by comparison of fragment size distributions

The methodology behind building a reference set for within-sample testing can be readily generalized for different types of data. However, this is under the assumption that it is possible to discretize these data in a manner that is analogous to the read coverage. We noted that the fragment size statistics itself can even at a sub-chromosomal scale, distinguish positive from negative samples. The richest representation of the fragment size statistics is to consider it as a probability distribution of fragment sizes within a region, denoting a probability of observing a certain fragment size. Hence when discretizing the genome into regions of a set size, each region maps to a corresponding probability vector. As described in Methods 2.2, we capped these distribution at 300 bp, such that only the range [0, 300] is considered.

Integrating this data representation requires a number of changes within the within-sample testing methodology. When constructing the reference set, it is no longer feasible to calculate the Euclidean distance between regions. Instead an appropriate distance measure is required to compare probability distributions, for this purpose we chose the Jensen-Shannon Divergence[2] (JSD) distance[3]. The JSD is a symmetrized generalization of the Kullback-Leibler Divergence[1] (KLD), and can be used as a distance metric.

$$D_{\mathbf{KL}}(P \parallel Q) = \sum_{x \in \mathcal{X}} P(x) \log \left(\frac{P(x)}{Q(x)} \right)$$ [1]

$$D_{\mathbf{JS}}(P \parallel Q) = \frac{1}{2} \left(D_{\mathbf{KL}}(P \parallel M) + D_{\mathbf{KL}}(Q \parallel M) \right), \textbf{Where } M = \frac{1}{2} \left(P + Q \right)$$ [2]

(M is the average of two distributions)

$$dist(P, Q) = \sqrt{D_{\mathbf{JS}}(P \parallel Q)}$$ [3]

The KLD was not appropriate for this purpose given that all regions are compared in a two-way fashion (each region is compared to all other regions on other chromosomes), since it is not symmetric it would fail to generate a correct reference set. A caveat that must be considered when utilizing the JSD, is that there must be no zero probabilities present within the compared distributions. We dealt with this issue by uniformly padding the distributions such that the previous zero values become extremely small probabilities. Computationally this probability vector-based approach is far costlier than when utilizing a single summary statistic per region.

With the reference set constructed a sample can be processed in an analogous manner as described for the summarized values. For example to compute the Z-score of a query sample region, we first combine the reference region distributions into a single combined distribution, and then calculate the JSD of the query region with respect to this combined distribution[1]. Next we obtain the mean and standard deviation by calculating the JSD of each reference region distribution with respect to the combined distribution. The region Z-score can then be derived from these three values (JSD query region distance with respect to the combined distribution, mean, and standard deviation).

We found that this approach, WcrFF^{FS_JSD}, which directly compares the fragment size distributions, was not effective to predict CNVs. In Figure S7 we show the average Z-scores across chromosome 21 for each of the 526 samples for regions of 1 Mb size, comparing WcrFF^{RC} to WcrFF^{FS_JSD}. It is clear that the divergence based method is not able to distinguish positive from negative samples. We believe that this bad performance can be explained by the high amount of noise present within the distributions, and that the JSD is too sensitive to handle this. We attempted to reduce the influence of noise by capping the distributions to smaller ranges (e.g. [125, 200]), or by smoothing the distributions, but this did not improve performance. If we consider the performance of the fragment size mean we observed that it improved as the region size increased (peaking at 10 Mb). It is possible that the divergence based approach would perform better with higher coverage (for more accurate distribution reconstruction) and larger region sizes. Additionally, it may be worthwhile to investigate other distribution distance metrics such as the Wasserstein distance (Earth mover's distance), although initial experiments were not promising.

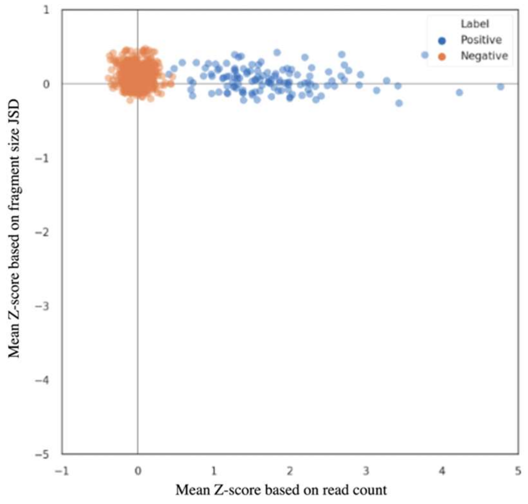

Figure S7. The average Z-score of all regions (1 Mb region size) on chromosome 21 for all 526 samples for WcrFF^{RC} compared to WcrFF^{FS_JSD}.
